# Author Correction: Paclitaxel plus carboplatin and durvalumab with or without oleclumab for women with previously untreated locally advanced or metastatic triple-negative breast cancer: the randomized SYNERGY phase I/II trial

**DOI:** 10.1038/s41467-023-44071-8

**Published:** 2023-12-12

**Authors:** Laurence Buisseret, Delphine Loirat, Philippe Aftimos, Christian Maurer, Kevin Punie, Véronique Debien, Paulus Kristanto, Daniel Eiger, Anthony Goncalves, François Ghiringhelli, Donatienne Taylor, Florent Clatot, Tom Van den Mooter, Jean-Marc Ferrero, Hervé Bonnefoi, Jean-Luc Canon, Francois P. Duhoux, Laura Mansi, Renaud Poncin, Philippe Barthélémy, Nicolas Isambert, Zoë Denis, Xavier Catteau, Roberto Salgado, Elisa Agostinetto, Evandro de Azambuja, Françoise Rothé, Ligia Craciun, David Venet, Emanuela Romano, John Stagg, Marianne Paesmans, Denis Larsimont, Christos Sotiriou, Michail Ignatiadis, Martine Piccart-Gebhart

**Affiliations:** 1 Université Libre de Bruxelles (ULB), Hôpital Universitaire de Bruxelles (HUB), Institut Jules Bordet, 1070 Brussels, Belgium; 2 Medical Oncology Department, Institut Curie, 75005 Paris, France; 3 Department I of Internal Medicine, Center for Integrated Oncology Aachen Bonn Cologne Duesseldorf, University of Cologne, 52074 Cologne, Germany; 4 Department of General Medical Oncology and Multidisciplinary Breast Unit, Leuven Cancer Institute, University Hospitals Leuven, 3000 Leuven, Belgium; 5 Medical Oncology Department, Institut Paoli-Calmettes, 13274 Marseille, France; 6 Medical Oncology Department, Centre Georges-François Leclerc, 21000 Dijon, France; 7 Department of Oncology, CHU-UCL-Namur - Site Sainte-Elisabeth, 5000 Namur, Belgium; 8 Medical Oncology Department, Centre Henri Becquerel, 76038 Rouen, France; 9 Department of Oncology, GZA Ziekenhuizen Campus Sint-Augustinus, 2610 Antwerp, Belgium; 10 Department of Oncology, Centre Antoine Lacassagne, 06189 Nice, France; 11 Medical Oncology Department, Institut Bergonié, 33000 Bordeaux, France; 12 Department of Oncology-Hematology, Grand Hôpital de Charleroi - Site Notre Dame, 6000 Charleroi, Belgium; 13 Medical Oncology Department, Cliniques Universitaires Saint-Luc (UCLouvain), 1200 Brussels, Belgium; 14 Department of Oncology, CHU Besançon - Hôpital Jean Minjoz, 25030 Besancon, France; 15 Medical Oncology Department, Clinique Saint-Pierre, 1340 Ottignies-Louvain-la-Neuve, Belgium; 16 Medical Oncology Department, Institut de Cancérologie Strasbourg Europe (ICANS), 67000 Strasbourg, France; 17 Medical Oncology Department, CHU Poitiers, 86000 Poitiers, France; 18 CurePath Laboratory (CHU Tivoli, CHIREC), 6040 Jumet, Belgium; 19 Department of Pathology, GZA-ZNA Hospitals, 2610 Antwerp, Belgium; 20 Centre for Cancer Immunotherapy, Medical Oncology Department, INSERM U932, Institut Curie, PSL Research University, 75005 Paris, France; 21 Centre de Recherche du Centre Hospitalier de l’Université de Montréal, Faculté de Pharmacie et Institut du Cancer de Montréal, Montréal, QC 11290 Canada

**Keywords:** Breast cancer, Breast cancer, Cancer immunotherapy

Correction to: *Nature Communications* 10.1038/s41467-023-42744-y, published online 02 November 2023

In the “Discussion” section of the original manuscript, the authors reported information and data related to an unpublished work (*Metoikidou C. and colleagues)*. Considering the confidential nature of these data, it has been decided to remove the related paragraph from the “Discussion” section. The original text has been replaced with the following statement:

“*In an ongoing analysis by single-cell RNA sequencing on tumors from a subset of patients from the SYNERGY trial, Metoikidou C. and colleagues observed distinct transcriptomic and TCR-clonal patterns in responders vs. non-responder patients and between treatment arms (personal communication). These analyses will shed light on the immune parameters associated with outcome to chemo-immunotherapy.”*

These changes have been implemented in the PDF and HTML versions of the Article.

