# Supplementary material for: Paclitaxel plus carboplatin and durvalumab with or without oleclumab for women with previously untreated locally advanced or metastatic triple-negative breast cancer: the randomized SYNERGY phase I/II trial
Source: Nat Commun. 2023 Nov 2;14:7018. doi: 10.1038/s41467-023-42744-y (PMC10622534; doi:10.1038/s41467-023-42744-y)
Supplement: Supplementary file 1 — Supplementary Information [file 41467_2023_42744_MOESM1_ESM.pdf]

Supplementary information

Figures:

- Supplementary Fig.1: Study Design
- Supplementary Fig.2: Subgroup analyses of Clinical Benefit Rate
- Supplementary Fig.3: Survival analyses according to PD-L1 status
- Supplementary Fig.4: survival analyses according to PD-L1 status in Arm A and B
- Supplementary Fig.5: Survival analyses according to CD73 status
- Supplementary Fig.6: flow diagram of evaluable tumor samples at baseline and week 3
- Supplementary Fig.7: Survival analyses according to str-TILs subgroup (low≤10% vs. high >10%) in arm A and B and str-TIL levels according to PD-L1 status and CPS
- Supplementary Fig.8: CBR and survival outcomes according to PD-L1 CPS
- Supplementary Fig.9: survival analyses according to CPS in Arm A and B
- Supplementary Fig.10: Multivariate analyses of clinical benefit rate, PFS and OS
- Supplementary Fig. 11: Baseline and week 3 PD-L1 combined positive score (CPS)
- Supplementary Fig.12: CD73 expression

Tables:

- Supplementary Table 1: Baseline characteristics and outcomes of patients enrolled in phase I part of the study.
- Supplementary Table 2: Tumor sample sites - prospective biomarker analyses
- Supplementary Table 3: Baseline characteristics of long responders
- Supplementary Table 4: Adverse events of special interest encoded per Preferred Term (MedRa coding) per arm, per number of patient, number of event of any grade and number of event of grade 3
- Supplementary Table 5: stromal TILs and PD-L1 expression at baseline and week 3
- Supplementary Table 6: Tumor sample sites - exploratory restrospective biomarker analyses
- Supplementary Table 7: Stromal TILs and PD-L1 CPS categories between baseline and week 3
- Supplementary Table 8: CD73 histological score – exploratory cohort

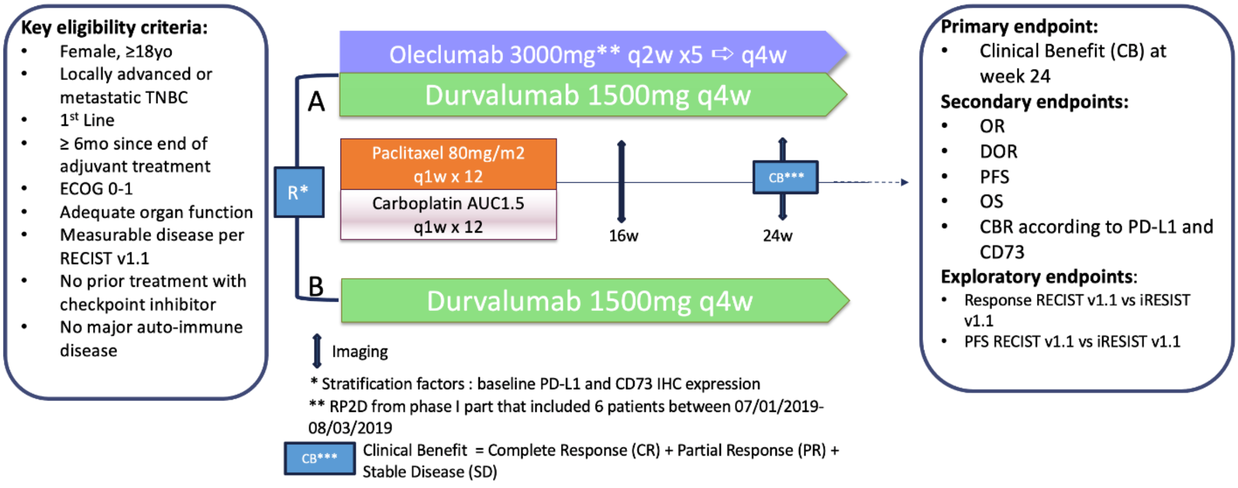

Supplementary Fig.1. Study design.

The SYNERGY trial is a multicenter, open-label, randomized (1:1 ratio) trial. Patients were randomized between two arms to be treated with 12 intravenous administrations of weekly 80mg/m^2 paclitaxel and AUC 1.5 of carboplatin in combination with 1500mg durvalumab once every 4 weeks with 3000 mg with or without oleclumab every 2 weeks (q2w) for 5 administrations and then q4w.

Abbreviations: yo: years old; mo: months; ECOG: Eastern Cooperative Oncology Group; R: randomization; RP2D: recommended for phase II dose; IHC: immunohistochemistry; w: week; OR objective response; DOR: duration of response; PFS: progression-free survival; OS: overall survival; CBR: clinical benefit rate.

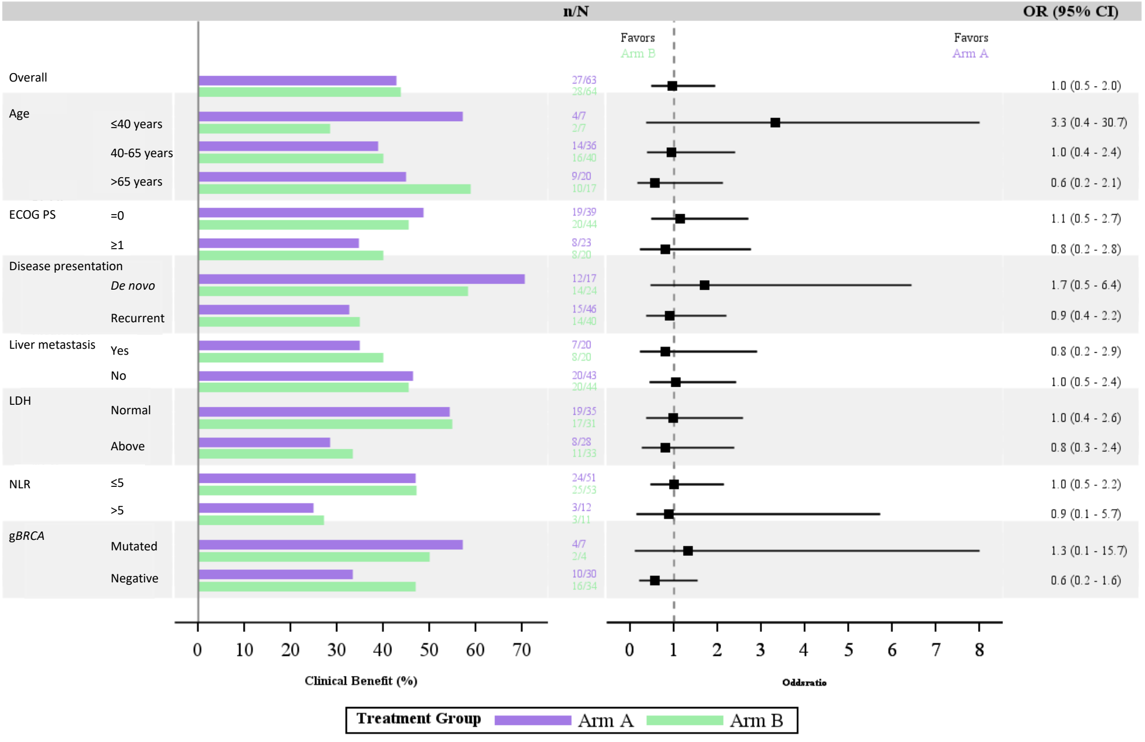

Supplementary Fig.2. Subgroup analyses of clinical benefit rate.
Percentage of clinical benefit is indicated for each arm per subgroup. The number of patients in each subgroup/arm is indicated. The odds-ratio is calculated as odds of clinical benefit in Arm A/odds of clinical benefit in arm B. The p-value is based on two-sided Fisher’s exact test. Abbreviations: NLR: neutrophils-to-lymphocytes ratio.

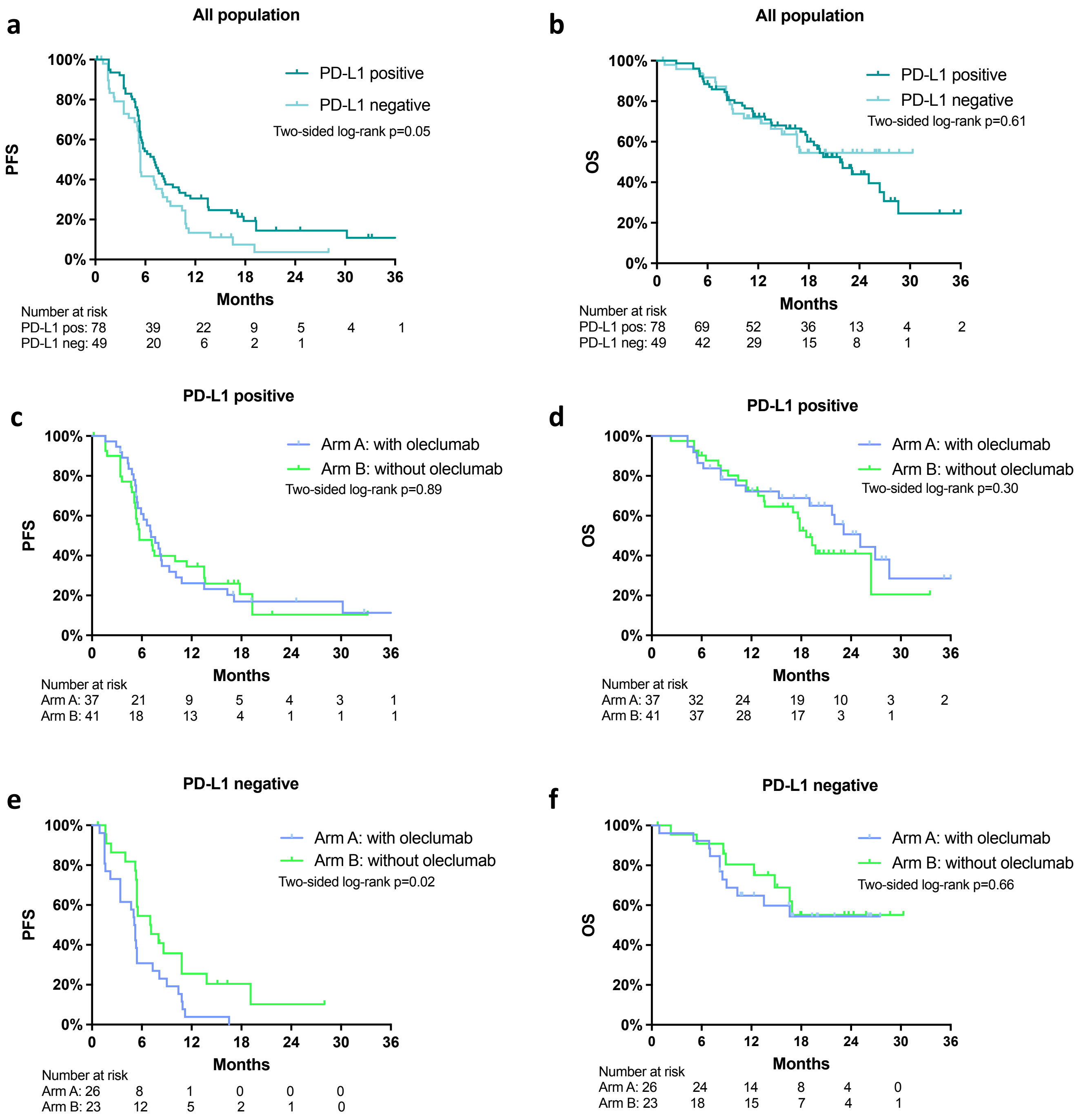

Supplementary Fig.3. Survival analyses according to PD-L1 status.

a. Kaplan-Meier estimates of PFS by RECIST v1.1 in the overall study population according to PD-L1 status.
b. Kaplan Meier estimates of OS in all population according to PD-L1 status.
c. Kaplan-Meier estimates of PFS by RECIST v1.1 in PDL-L1 positive population according to treatment arm.
d. Kaplan Meier estimates of OS in PDL-L1 positive population according to treatment arm.
e. Kaplan-Meier estimates of PFS by RECIST v1.1 in PD-L1 negative population according to treatment arm.
f. Kaplan Meier estimates of OS in PD-L1 negative population according to treatment arm.

Two-sided log-rank test was used for all survival comparisons.

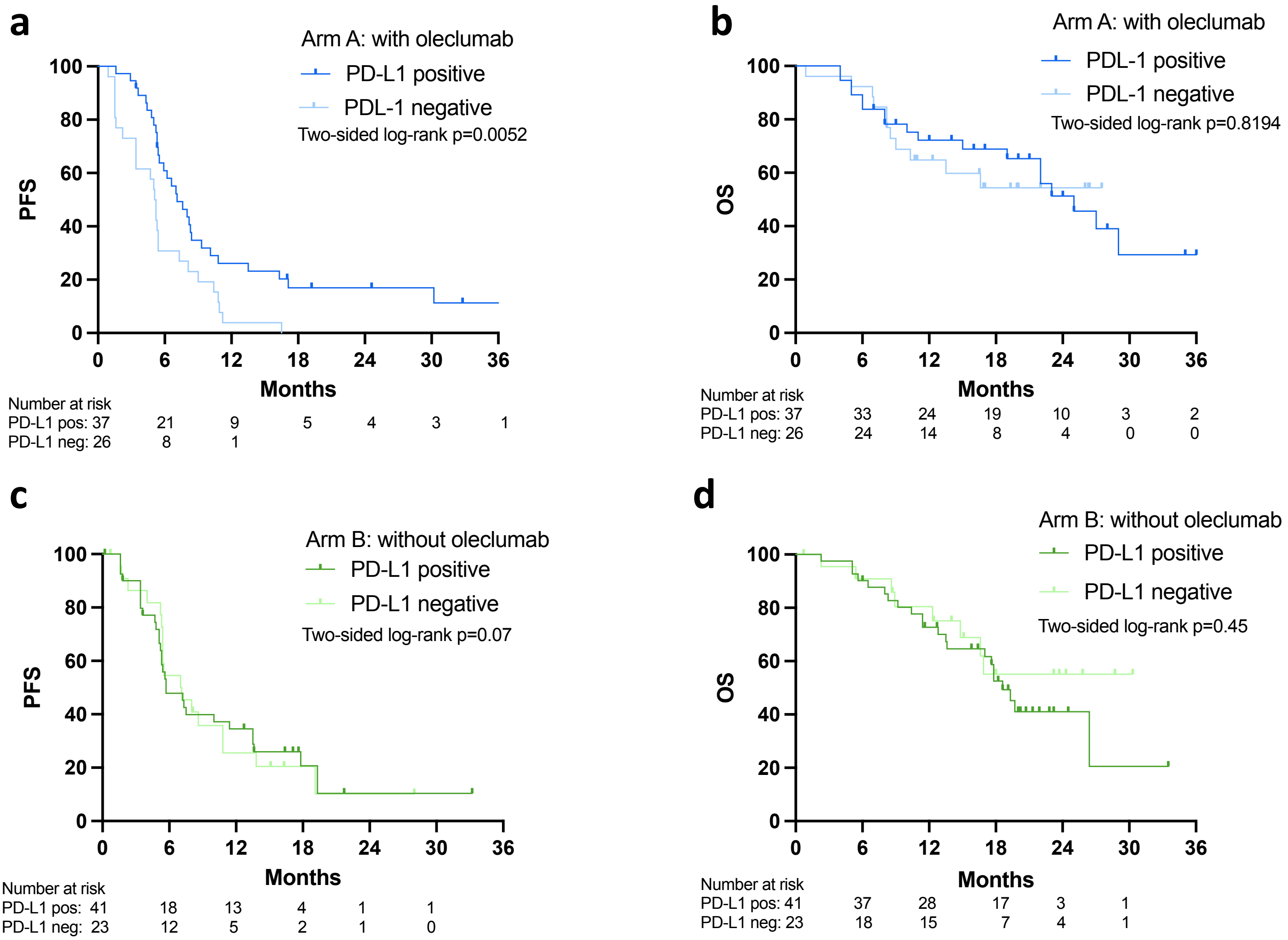

Supplementary Fig.4. Survival analyses according to PD-L1 status in arm A and B.

a. Kaplan-Meier estimates of progression-free survival (PFS) by RECIST v1.1 according to PD-L1 status in arm A (with oleclumab).
b. Kaplan-Meier estimates of overall survival (OS) according to PD-L1 status in arm A (with oleclumab).
c. Kaplan-Meier estimates of PFS by RECIST v1.1 according to PD-L1 status in arm B (without oleclumab).
d. Kaplan Meier estimates of OS according to PD-L1 status in arm B (without oleclumab).

Two-sided log-rank test was used for all survival comparisons.

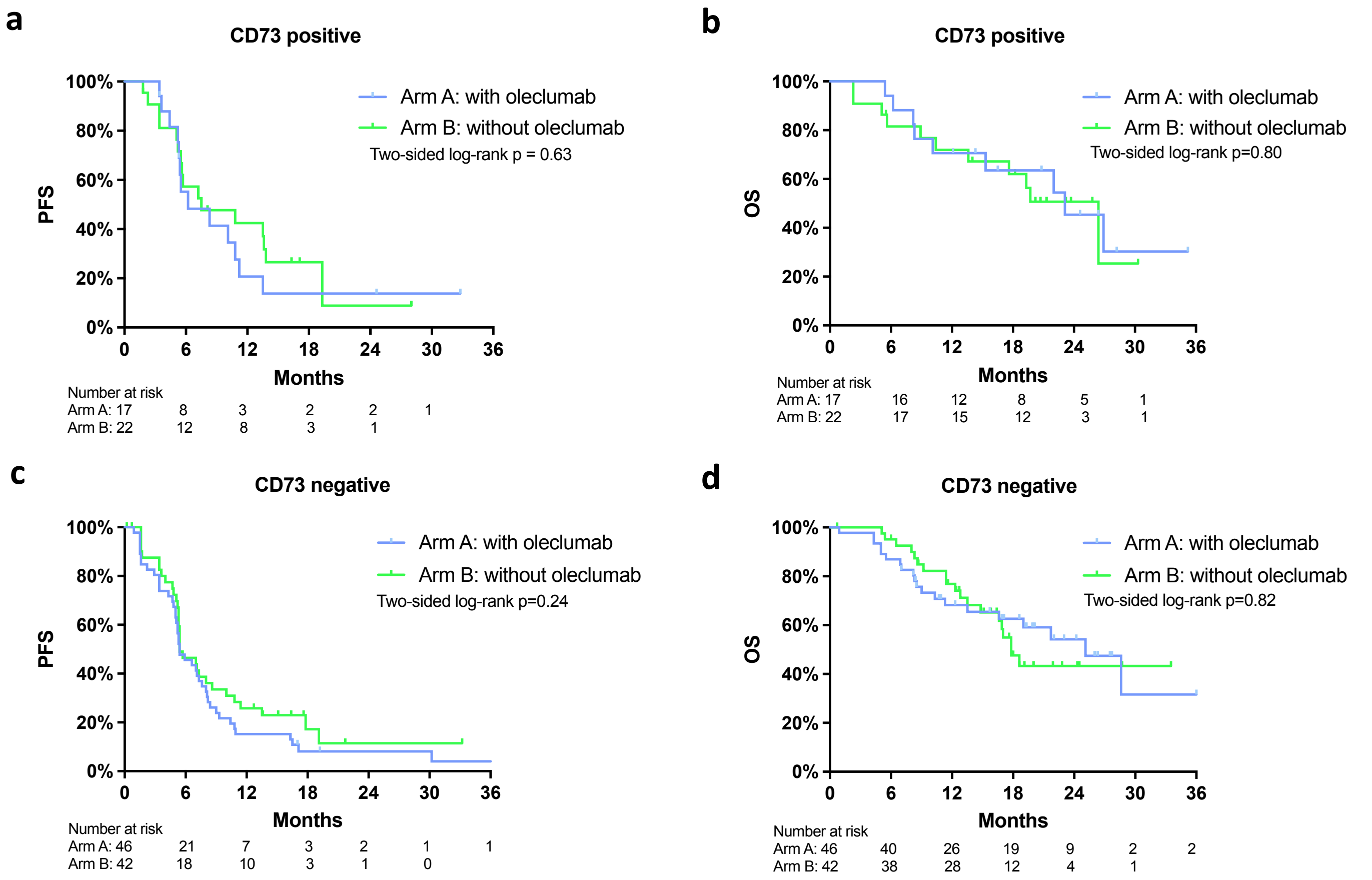

Supplementary Fig.5. Survival analyses according to CD73 status.

a. Kaplan-Meier estimates of PFS by RECIST v1.1 in CD73 positive population according to treatment arm.

b. Kaplan Meier estimates of OS in CD73 positive population according to treatment arm.

c. Kaplan-Meier estimates of PFS by RECIST v1.1 in CD73 negative population according to treatment arm.

d. Kaplan Meier estimates of OS in CD73 negative population according to treatment arm.

Two-sided log-rank test was used for all survival comparisons.

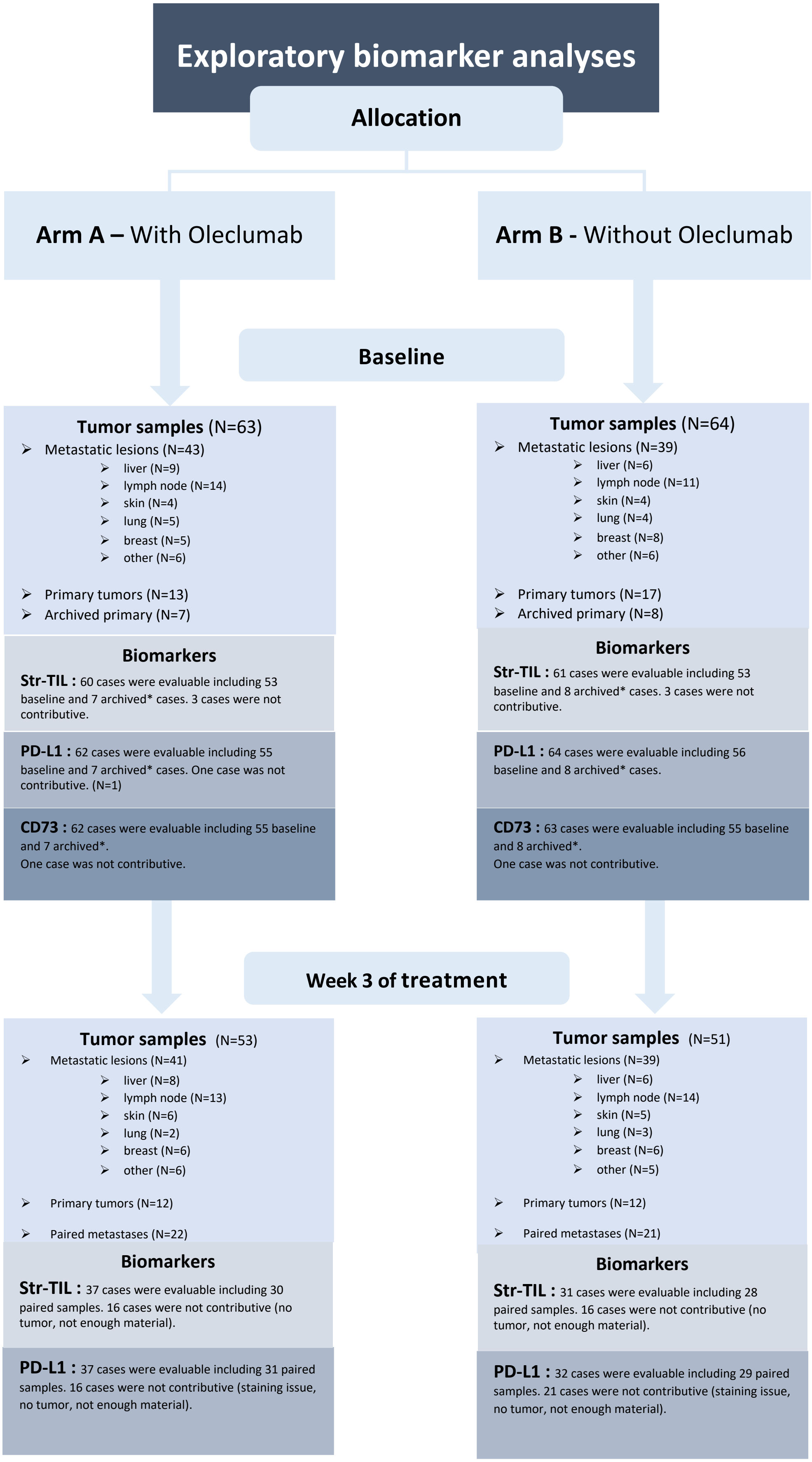

* Archived samples were excluded from the baseline retrospective biomarker analyses.

Supplementary Fig.6. Flow diagram of collected and analyzed tumor samples at baseline and week 3.

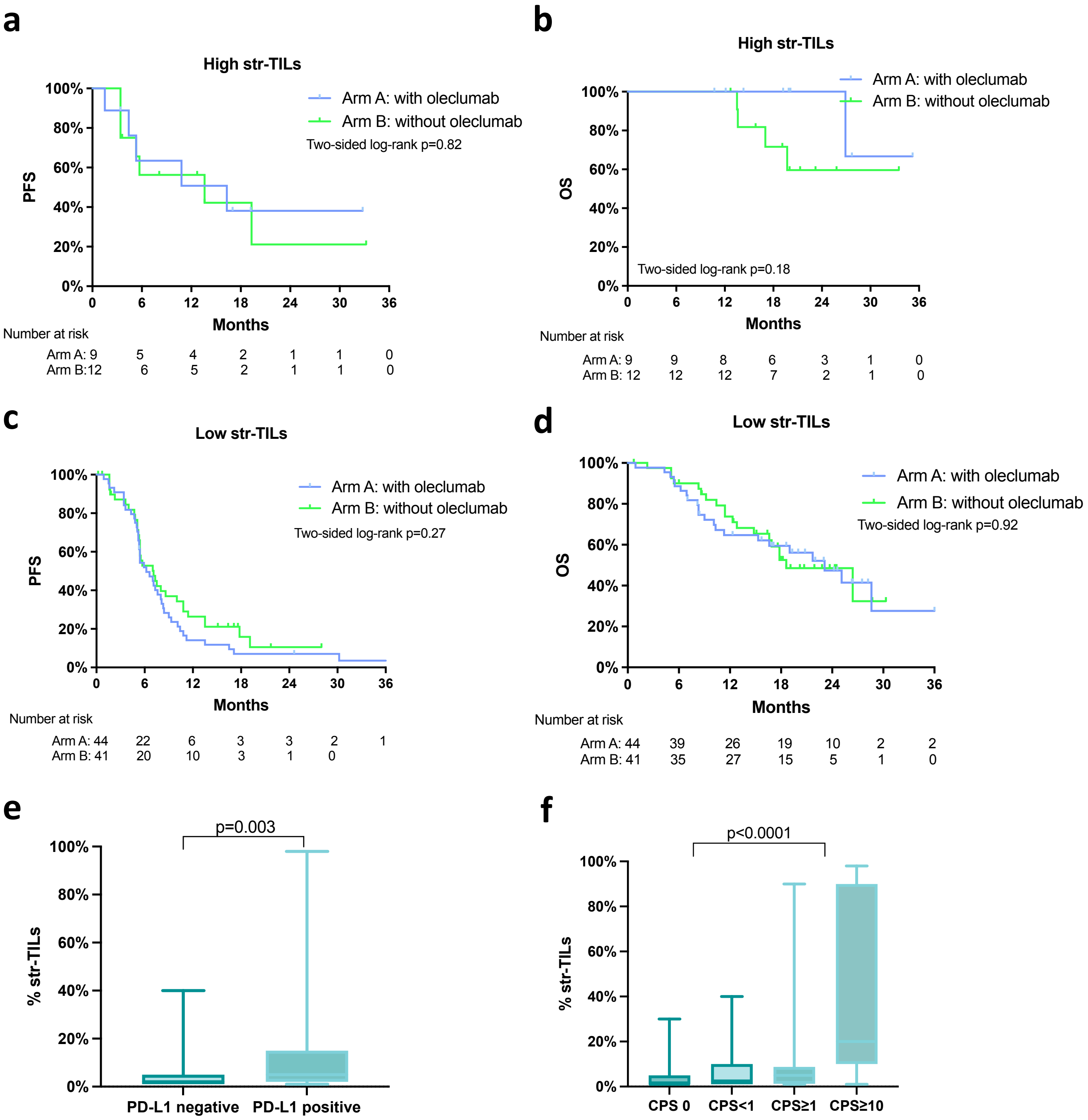

Supplementary Fig.7. Survival analyses according to baseline str-TILs subgroup (low≤10% vs. high >10%) in arm A and B and str-TIL levels according to PD-L1 status and CPS.

a. Kaplan-Meier estimates of progression-free survival (PFS) in str-TILs high (>10%) subgroup per treatment arm. Two-sided log-rank test.
b. Kaplan-Meier estimates of PFS in str-TILs high (>10%) subgroup per treatment arm. Two-sided log-rank test.
c. Kaplan-Meier estimates of PFS in str-TILs low (≤10%) subgroup per treatment arm. Two-sided log-rank test.
d. Kaplan-Meier estimates of OS in str-TILs low (≤10%) subgroup per treatment arm. Two-sided log-rank test.
e. Baseline Str-TILs according to prospective PD-L1 status (PD-L1 negative (N=39) and PD-L1 positive (N=68). Wilcoxon non-parametric test.
f. Baseline Str-TILs according to exploratory PD-L1 CPS (CPS 0 (N=27), CPS<1 (N=44), CPS ≥1 (N=16) and CPS ≥10 (N=19). Wilcoxon non-parametric test.

Source data are provided as a Source Data file.

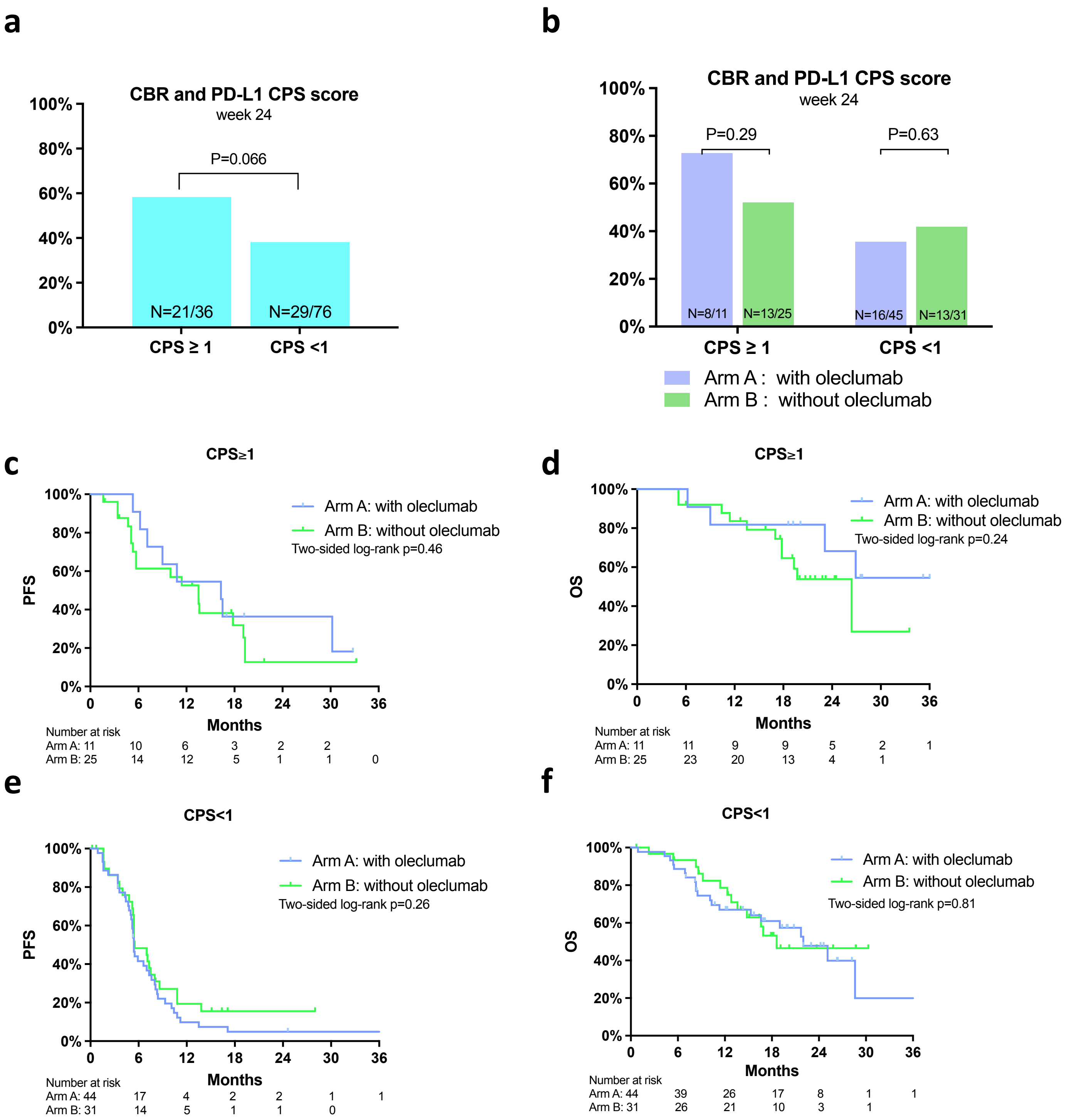

Supplementary Fig.8. CBR and survival outcomes according to PD-L1 CPS.

a. CBR according to PD-L1 combined positive score (CPS) in the whole trial population. One-sided Fisher's exact test.
b. CBR according to PD-L1 CPS in arm A (with oleclumab) and arm B (without oleclumab). Two-sided Fisher's exact test. No type-1 error adjustments for multiple comparisons.
c. Progression –free survival (PFS) in CPS positive according to treatment arm.
d. Overall survival (OS) in CPS positive according to treatment arm.
e. PFS in CPS negative according to treatment arm.
f. OS in CPS negative according to treatment arm.

Two-sided log-rank test was used for all survival comparisons.

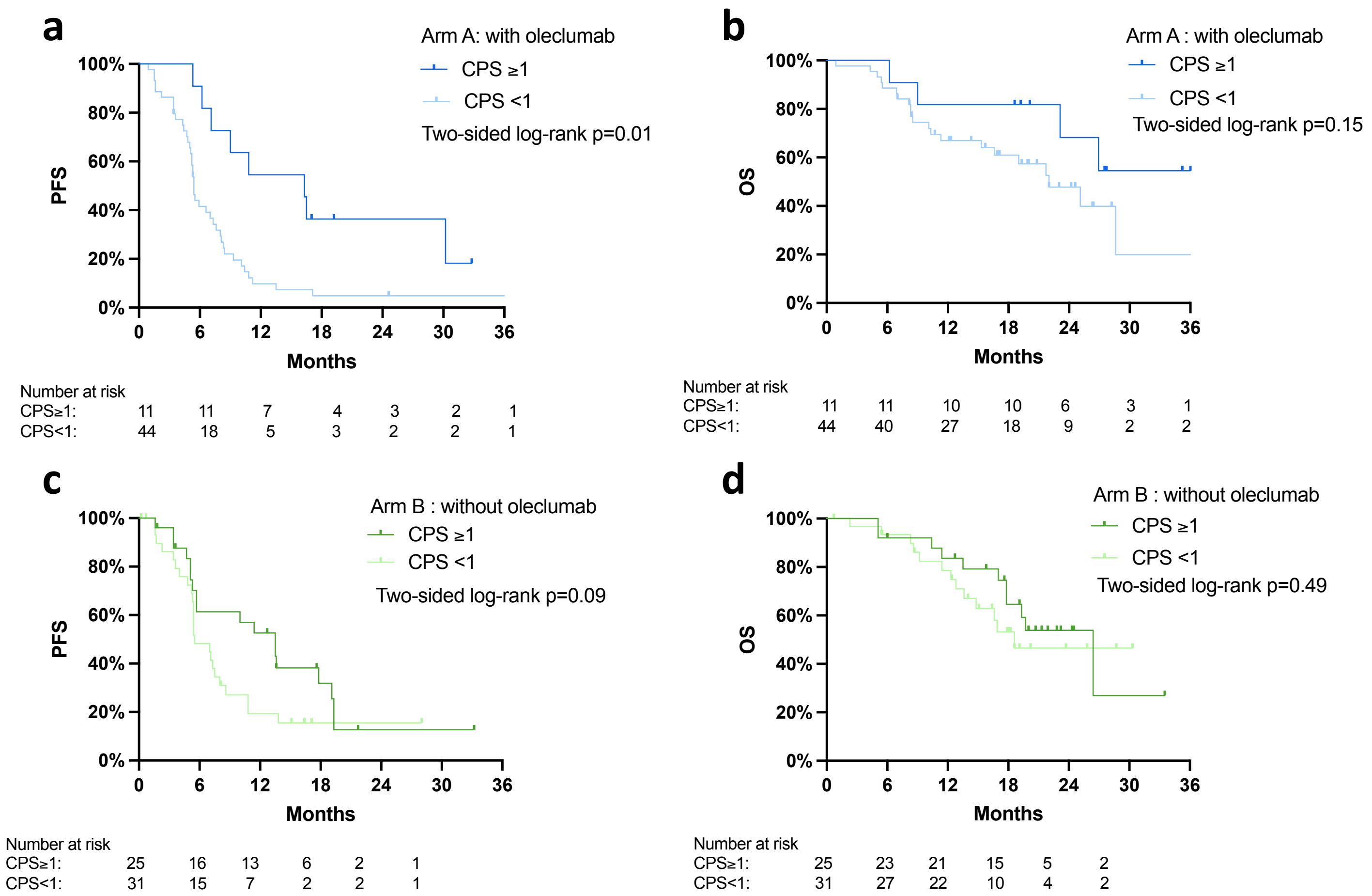

Supplementary Fig.9. Survival analyses according to PD-L1 CPS in Arm A and Arm B.

a. Kaplan-Meier estimates of PFS according to CPS in arm A (with oleclumab).
b. Kaplan-Meier estimates of OS according to CPS in arm A (with oleclumab).
c. Kaplan-Meier estimates of PFS according to CPS in arm B (without oleclumab).
d. Kaplan-Meier estimates of OS according to CPS in arm B (without oleclumab).

Two-sided log-rank test was used for all survival comparisons.

a

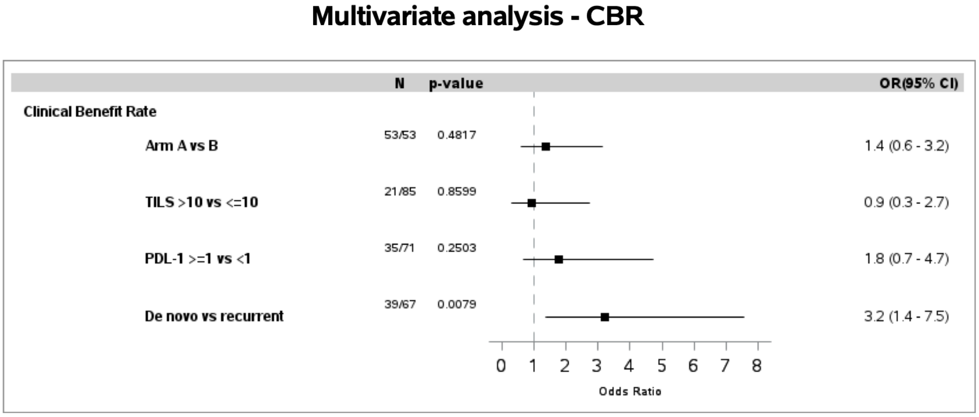

b

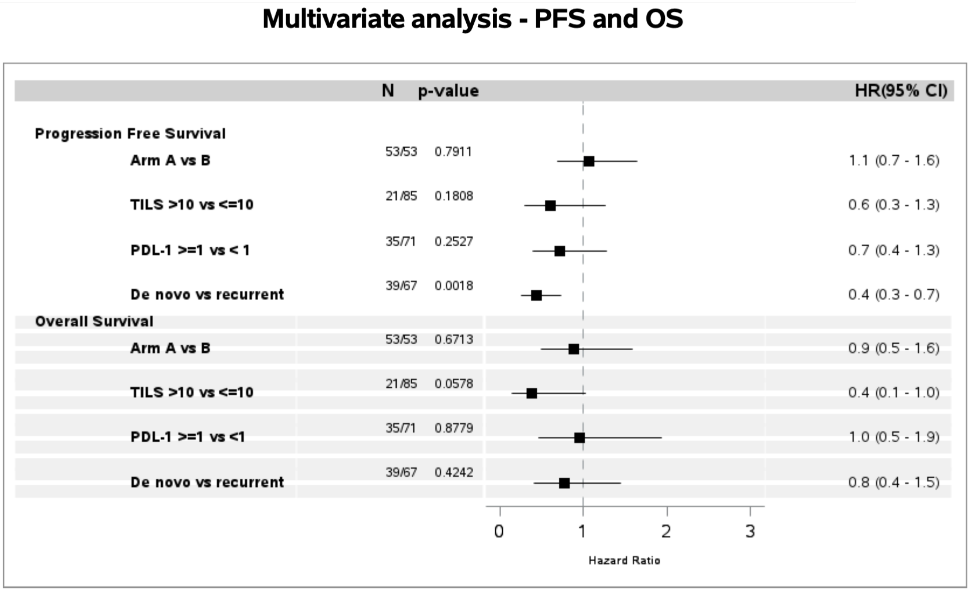

Supplementary Fig.10. Multivariate analyses of clinical benefit rate, PFS and OS.

a. Multiple logistic regression results of clinical benefit explained by arm, TILs, PD-L1 CPS and disease presentation. The odds-ratios (OR, center square) are calculated for each variable given that the other variables are set at the reference values (Arm B, TILs ≤10%, CPS<1, recurrent). OR>1 indicates that the variable is associated with clinical response.
b. Multiple Cox regression results of PFS/OS explained by arm, TILs, PD-L1 CPS and disease presentation. The hazard ratios (HR, center square) are calculated for each variable given that the other variables are set at the reference values (Arm B, TILs ≤10%, CPS<1, recurrent). HR <1 indicates that the variable is associated with better prognosis.

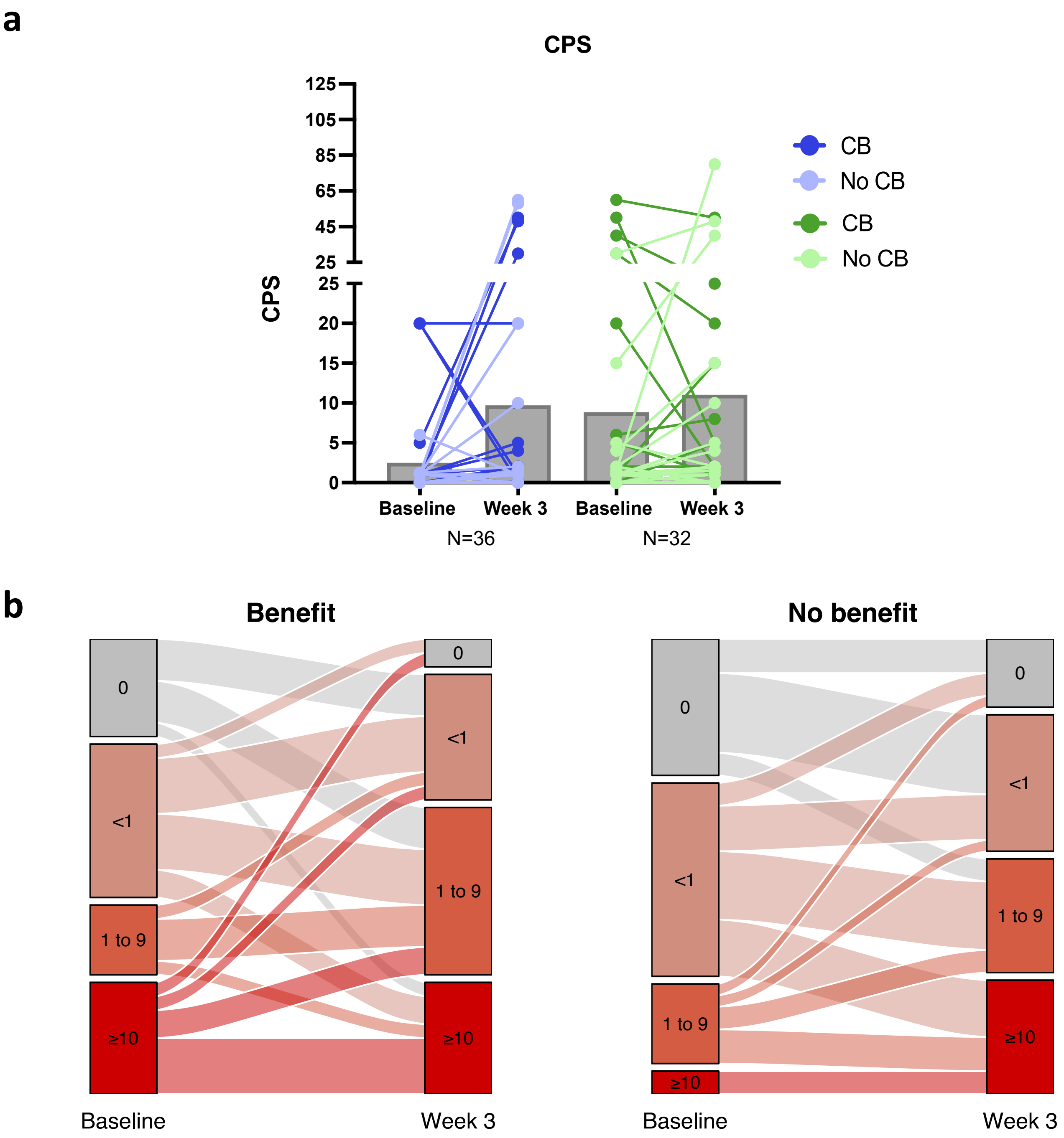

Supplementary Fig. 11. Baseline and week 3 PD-L1 combined positive score (CPS).

a. Dynamic of PD-L1 CPS between baseline and week 3 biopsies in arm A (with oleclumab) and in arm B (without oleclumab) (36 paired samples in arm A and 32 in arm B). Patients with clinical benefit (CB) at week 24 are represented in dark blue (arm A) and dark green (arm B)

b. Alluvial plots showing the transition of CPS between paired baseline and week 3 tumor samples in patients with clinical benefit at week 24 (n=28) and no benefit (n=34).

Source data are provided as a Source Data file.

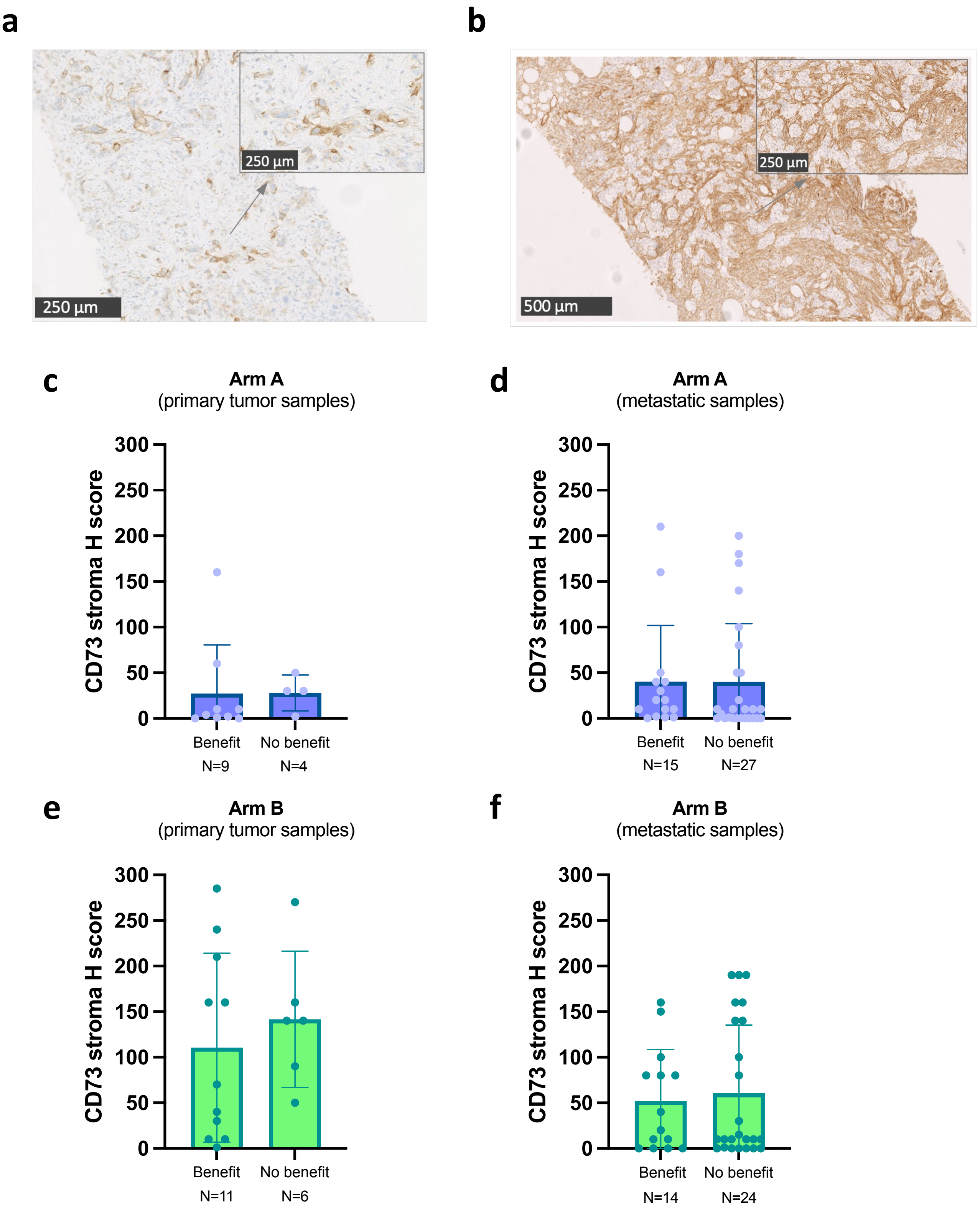

Supplementary Fig.12. CD73 expression.

a. Representative CD73 IHC staining showing CD73 expression on tumor cells.
b. Representative CD73 IHC staining showing CD73 expression on stroma cells.
c. CD73 stroma H scores in baseline primary tumors biopsies in Arm A (with oleclumab) according to clinical benefit at week 24.
d. CD73 stroma H scores in baseline metastatic tumor biopsies in Arm A (with oleclumab) according to clinical benefit at week 24.
e. CD73 stroma H scores in baseline primary tumor biopsies in arm B (without oleclumab) according to clinical benefit.
f. CD73 stroma H scores in baseline metastatic tumor biopsies in Arm B (without oleclumab) according to clinical benefit at week 24.

Source data are provided as a Source Data file.

Supplementary Table 1. **Baseline characteristics and outcomes of patients enrolled in phase I part of the study.**

Patient	1*	2	3	4	5*	6
Age (years)	≤65	≤65	≤65	≤65	>65	>65
Recurrent/de novo	Recurrent	Recurrent	Recurrent	Recurrent	Recurrent	Recurrent
Response at week 24	PR	PD	SD	PD	PR	PR
Grade 3/4 toxicities	Pneumonia + Febrile Neutropenia; Herpes Zoster; Fatigue; Pulmonary Embolism	Neutropenia	Neutropenia (3X)	Neutropenia (3X); dyspnea	Neutropenia (2X)	Deep vein thrombosis + Lung embolism;
PFS	21.7	1.7	7.3	4.3	44.1	11
OS	47.4	2.7	16.9	10.7	45.9	27.3
ECOG	0	0	0	1	1	1
BRCA status	Negative	Negative	UKN	UKN	Negative	Negative
Disease-free interval	75 months	14 months	24 months	23 months	42 months	62 months
Prior chemotherapy						
Anthracyclines	yes	yes	yes	yes	yes	yes
Taxanes	yes	yes	yes	yes	yes	yes
Carboplatin	no	no	no	no	yes	no
Metastatic sites, number	2	5	3	5	5	1
Metastatic sites (targets and non targets)						
Liver	-	yes	yes	-	yes	-
Bone	-	yes	yes	-	yes	-
Lung	yes	yes	-	yes	yes	yes
Lymph nodes	yes	yes	-	yes	yes	-
Others	-	yes	yes	yes	yes	-
LDH	257 (>UNL)	329 (>UNL)	1754 (>UNL)	1463 (>UNL)	259 (>UNL)	166 (<UNL)
NLR	1.74	3.68	2.96	4.95	11.25	4.3

*Patient still under treatment

Abbreviations: PR: partial response, PD: progressive disease, SD: sable disease, X: times, UKN: unknown, LDH: lactate dehydrogenase, UNL: upper normal limit, NLR: neutrophils-to-lymphocytes ratio

Supplementary Table 2. Tumor sample sites - prospective biomarker analyses.

Tissue/organ	N	PD-L1 CPS ≥ 1	PD-L1 CPS <1	CD73 Hscore ≥ 1	CD73 Hscore <1
**Primary tumor**	45				
De novo	30	19 (63.3%)	11 (36.7%)	5 (16.7%)	25 (83.3%)
**Archived**	15	9 (60%)	6 (40%)	6 (40%)	9 (60%)
**Metastatic lesi**	82				
**Breast**	13	7 (53.8%)	6 (46.2%)	7 (53.8%)	6 (46.2%)
**Brain**	1	1 (100%)	0 (0%)	0 (0%)	1 (100%)
**Chest Wall**	5	3 (60%)	2 (40%)	1 (20%)	4 (80%)
**Liver**	15	11 (73.3%)	4 (26.7%)	6 (40%)	9 (60%)
**Lung**	9	6 (66.7%)	3 (33.3%)	3 (33.3%)	6 (66.7%)
**Lymph node**	25	14 (56%)	11 (44%)	7 (28%)	18 (72%)
**Peritoneal**	1	0 (0%)	1 (100%)	1 (100%)	0 (0%)
**Skin**	8	6 (75%)	2 (25%)	1 (12.5%)	7 (87.5%)
**Uterus**	1	0 (0%)	1 (100%)	1 (100%)	0 (0%)
**Other**	2	1 (50%)	1 (50%)	1 (50%)	1 (50%)
**Not reported**	2	1 (100%)	1 (100%)	0 (0%)	2 (100%)
**Total**	127	78	49	39	88

Supplementary Table 3. Baseline characteristics of long responders.

Baseline characteristics of long responders (PFS≥12 months), and those with progressive disease before 12 months. Seven subjects who discontinued the treatment before 12 months without any PD RECIST evaluation are excluded. All tests are two-sided. No type-1 error adjustments for multiple comparisons. Wilcoxon non-parametric test is used to analyze continuous variables. Fisher's exact test is used to analyze categorical variables.

Characteristics	Long Responders<br>PFS ≥ 12 months<br>(N=28)	Other patients<br>PFS < 12 months<br>(N=92)	p-value
**Age, in years**			
Median (IQR)	59.0 (53.0, 69.5)	57.0 (47.0, 67.0)	0.26
<40	1 (3.6%)	12 (13.0%)	0.43
40-65	18 (64.3%)	53 (57.6%)	
>65	9 (32.1%)	27 (29.3%)	
**ECOG Performance Status**			
0	20 (71.4%)	57 (62.6%)	0.62
1	8 (28.6%)	33 (36.3%)	
2	0 (0.0%)	1 (1.1%)	
Missing	0	1	
**Disease presentation**			
De novo metastatic	18 (66.7%)	20 (21.7%)	<0.0001
Recurrent metastatic	10 (35.7%)	72 (78.3%)	
**Metastatic sites, number**			
<2	7 (25.0%)	18 (19.6%)	0.60
≥2	21 (75.0%)	74 (80.4%)	
**Metastatic sites (targets)**			
Liver	7 (25.0%)	32 (34.8%)	0.36
Bone	6 (21.4%)	22 (23.9%)	1
Lung	13 (46.4%)	42 (45.7%)	1
Lymph nodes	16 (57.1%)	52 (56.5%)	1
Others (skin, peritoneum, …)	11 (39.3%)	49 (53.3%)	0.28
**Germline *BRCA* mutational status**			
BRCA1/2 mutation	2 (7.1%)	8 (8.7%)	0.45
Absence of mutation	12 (42.9%)	49 (53.3%)	
Unknown	14 (50%)	35 (38.1%)	
**LDH**			
≤UNL	19 (67.9%)	50 (54.3%)	0.05
>UNL	9 (32.1%)	42 (45.7%)	
**NLR**			
≤5	24 (85.7%)	73 (79.3%)	0.59
>5	4 (14.3%)	19 (20.7%)	
**PD-L1 status** (baseline, at randomization)			
Negative (<1%)	12 (42.9%)	43 (46.7%)	0.03
Positive (≥1%)	16 (57.1%)	49 (53.3%)	
**CD73 status** (baseline, at randomization)			
Negative (<1%)	19 (67.9%)	65 (70.7%)	0.23
Positive (≥1%)	9 (32.1%)	27 (29.3%)	

Abbreviations: IQR: interquartile range; ECOG: Eastern Cooperative Oncology Group; LDH: Lactate dehydrogenase; NLR: neutrophils to lymphocytes ratio; PD-L1: Programmed cell Death Ligand 1, UNL: upper normal limit.

Supplementary Table 4. Adverse events of special interest encoded per Preferred Term (MedRa coding) per arm, per number of patient (a), number of event of any grade (b) and number of event of grade 3 (c).

	Number of patients	
AESI Preferred Term	A	B
Diarrhoea	22	26
Alopecia	20	18
Pruritus	14	6
Rash	12	8
Alanine aminotransferase decreased	7	6
Hypothyroidism	7	6
Dermatitis acneiform	6	6
Hepatic cytolysis	5	1
Hyperthyroidism	5	3
Arthralgia	4	0
Aspartate aminotransferase increased	3	3
Lymphoedema	3	0
Rash maculo-papular	3	1
Erythema	2	2
Gamma-glutamyltransferase increased	2	0
Hypersensitivity	2	1
Lipase increased	2	1
Psoriasis	2	0
Pulmonary embolism	2	0
Pyrexia	2	0
Vitiligo	2	0
Acute myocardial infarction	1	0
Adrenal insufficiency	1	1
Alopecia areata	1	0
Amylase increased	1	0
Arthritis	1	0
Blood creatine increased	1	0
Capillary permeability	1	0
Cholestasis	1	0
Deep vein thrombosis	1	0
Dermatitis	1	0
Dermatitis exfoliative generalised	1	1
Drug-induced liver injury	1	3
Dyspnoea	1	0
Epistaxis	1	0
Erythrosis	1	0
Eyelid rash	1	0
Face oedema	1	0
Fungal skin infection	1	0
Haemolytic anaemia	1	0
Hand dermatitis	1	0
Hepatocellular injury	1	0
Herpes virus infection	1	0
Hypophysitis	1	0
Immune-mediated hepatitis	1	0
Interstitial lung disease	1	1
Lung disorder	1	0
Lymphocyte count decreased	1	0
Myalgia	1	2
Nausea	1	0
Oedema peripheral	1	1
Pelvic venous thrombosis	1	0
Pulmonary microemboli	1	0
Rash erythematous	1	0
Renal failure	1	0
Rosacea	1	0
Abdominal pain upper	0	1
Acne	0	1
Aphasia	0	1
Colitis	0	3
Dyspnoea exertional	0	1
Eczema	0	1
Haematoma	0	1
Infusion related reaction	0	1
Lip dry	0	1
Localised oedema	0	1
Neutropenia	0	1
Pancreatitis	0	1
Rash pruritic	0	1
Rash pustular	0	1
Urticaria	0	1

	Number of event, any grade	
AESI Preferred Term	A	B
Diarrhoea	29	39
Alopecia	20	18
Pruritus	15	7
Rash	13	8
Alanine aminotransferase decreased	8	8
Hypothyroidism	7	6
Dermatitis acneiform	6	6
Hepatic cytolysis	6	1
Hyperthyroidism	6	3
Arthralgia	4	0
Aspartate aminotransferase increased	3	3
Lymphoedema	3	0
Rash maculo-papular	3	1
Erythema	1	5
Gamma-glutamyltransferase increased	2	0
Hypersensitivity	2	1
Lipase increased	2	1
Psoriasis	2	0
Pulmonary embolism	2	0
Pyrexia	2	0
Vitiligo	2	0
Acute myocardial infarction	1	0
Adrenal insufficiency	1	1
Alopecia areata	1	0
Amylase increased	1	0
Arthritis	1	0
Blood creatine increased	1	0
Capillary permeability	1	0
Cholestasis	1	0
Deep vein thrombosis	1	0
Dermatitis	1	0
Dermatitis exfoliative generalised	1	1
Drug-induced liver injury	2	3
Dyspnoea	1	0
Epistaxis	1	0
Erythrosis	1	0
Eyelid rash	1	0
Face oedema	1	0
Fungal skin infection	1	0
Haemolytic anaemia	1	0
Hand dermatitis	1	0
Hepatocellular injury	1	0
Herpes virus infection	1	0
Hypophysitis	1	0
Immune-mediated hepatitis	1	0
Interstitial lung disease	1	1
Lung disorder	1	0
Lymphocyte count decreased	1	0
Myalgia	1	2
Nausea	1	0
Oedema peripheral	1	1
Pelvic venous thrombosis	1	0
Pulmonary microemboli	1	0
Rash erythematous	1	0
Renal failure	1	0
Rosacea	1	0
Abdominal pain upper	0	1
Acne	0	1
Aphasia	0	1
Colitis	0	3
Dyspnoea exertional	0	1
Eczema	0	1
Haematoma	0	1
Infusion related reaction	0	1
Lip dry	0	1
Localised oedema	0	1
Neutropenia	0	1
Pancreatitis	0	1
Rash pruritic	0	1
Rash pustular	0	1
Urticaria	0	1

	Number of event, grade 3	
AESI Preferred Term	A	B
Haemolytic anaemia	1	0
Gamma-glutamyltransferase increased	1	0
Renal failure	1	0
Drug-induced liver injury	0	2
Adrenal insufficiency	1	0
Dermatitis	1	0
Immune-mediated hepatitis	1	0
Pulmonary embolism	2	0
Aspartate aminotransferase increased	0	1
Hypersensitivity	0	1
Alanine aminotransferase decreased	1	2
Erythema	1	0
Colitis	0	1
Pancreatitis	0	1

Supplementary Table 5. Stromal TILs and PD-L1 expression at baseline and week 3.

Characteristics	Arm A with oleclumab	Arm B without oleclumab	Total	Characteristics	Arm A with oleclumab	Arm B without oleclumab	Total
**Stromal TILs**							
**Baseline str-TILs**				**Week 3 str-TILs**			
N	53	53	106	N	37	31	68
≤10%	44 (83.0%)	41 (77.4%)	85 (80.2%)	≤10%	29 (78.4%)	22 (71.0%)	51 (75.0%)
>10%	9 (17.0%)	12 (22.6%)	21 (19.8%)	>10%	8 (21.6%)	9 (29.0%)	17 (25.0%)
Mean (SD)	12.9 (21.3)	20.3 (30.7)	16.6 (26.6)	Mean (SD)	14 (25.3)	18.5 (27.7)	16.1 (26.3)
Median (IQR)	5 (1-10)	4 (1-20)	5 (1-15)	Median (IQR)	3 (1-10)	1 (1-27.5)	2 (1-13.7)
**Primary tumors**							
≤10%	11 (91.7%)	13 (76.5%)	24 (82.8%)	≤10%	2 (66.7%)	1 (50%)	3 (60.0%)
>10%	1 (8.3%)	4 (23.5%)	5 (17.2%)	>10%	1 (33.3%)	1 (50%)	2 (40.0%)
**Metastatic lesions**							
≤10%	33 (80.5%)	28 (77.8%)	61 (79.2%)	≤10%	27 (79.4%)	21 (72.4%)	48 (76.2%)
>10%	8 (19.5%)	8 (22.2%)	16 (20.8%)	>10%	7 (20.6%)	8 (27.6%)	15 (23.8%)
**PD-L1 positivity**							
**Baseline PD-L1 positivity by cell type, all samples**				**Week 3 PD-L1 positivity by cell type, all samples**			
N	55	56	111	N	37	32	69
Immune cells	39 (70.9%)	38 (67.9%)	77 (69.4%)	Immune cells	30 (81.1%)	29 (90.6%)	59 (85.5%)
Tumor cells	3 (5.5%)	5 (8.9%)	8 (7.2%)	Tumor cells	18 (48.6%)	22 (68.7%)	40 (58.0%)
**Primary tumors**							
N	13	17	30	N	2	2	4
Immune cells	10 (76.9%)	16 (94.1%)	26 (86.7%)	Immune cells	1 (50.0%)	2 (100.0%)	3 (75.0%)
Tumor cells	1 (7.7%)	0 (0%)	1 (3.3%)	Tumor cells	2 (100.0%)	1 (50.0%)	3 (75.0%)
**Metastatic lesions**							
N	42	39	81	N	35	30	65
Immune cells	29 (69.0%)	22 (56.4%)	51 (63.0%)	Immune cells	29 (82.9%)	27 (90.0%)	56 (86.2%)
Tumor cells	2 (4.7%)	5 (12.8%)	7 (8.6%)	Tumor cells	16 (45.7%)	21 (70.0%)	37 (56.9%)
**PD-L1 CPS**							
**Baseline PD-L1 CPS**				**Week 3 PD-L1 CPS**			
N	55	56	111	N	37	32	69
0	15 (27.3%)	13 (23.2%)	28 (25.2%)	0	5 (13.5%)	3 (9.4%)	8 (11.6%)
<1	29 (52.7%)	18 (28.1)	47 (42.3%)	<1	15 (40.5%)	6 (18.8%)	21 (30.4%)
≥1 (<10)	3 (5.5%)	14 (25%)	17 (15.3%)	≥1 (<10)	8 (21.7%)	14 (43.7%)	22 (31.9%)
≥10	8 (14.5%)	11 (19.6%)	19 (17.1%)	≥10	9 (24.3%)	9 (28.1%)	18 (26.1%)
**Primary tumors**							
N	13	17	30	N	2	2	4
0	2 (15.4%)	1 (5.9%)	3 (10.0%)	0	0 (0.0%)	0 (0.0%)	0 (0.0%)
<1	8 (61.5%)	6 (35.3%)	14 (46.7%)	<1	1 (50.0%)	1 (50.0%)	2 (50.0%)
≥1 (<10)	2 (15.4%)	4 (23.5%)	6 (20.0%)	≥1 (<10)	0 (0.0%)	1 (50.0%)	1 (25.0%)
≥10	1 (7.7%)	6 (35.3%)	7 (23.3%)	≥10	1 (50.0%)	0 (0.0%)	1 (25.0%)
**Metastatic lesions**							
N	42	39	81	N	35	30	65
0	13 (31.0%)	12 (30.8%)	25 (30.9%)	0	5 (14.2%)	3 (10.0%)	8 (12.3%)
<1	21 (50.0%)	12 (30.8%)	33 (40.7%)	<1	14 (40.0%)	5 (16.7%)	19 (29.2%)
≥1 (<10)	1 (2.4%)	10 (25.6%)	11 (13.6%)	≥1 (<10)	8 (22.9%)	13 (43.3%)	21 (32.3%)
≥10	7 (16.7%)	5 (12.8%)	12 (14.8%)	≥10	8 (22.9%)	9 (30.0%)	17 (26.2%)

Supplementary Table 6. Tumor sample sites - exploratory restrospective biomarker analyses.

Tissue/organ	N	N	PD-L1, CPS <1	PD-L1, CPS ≥ 1	N	TILs <10%	TILs ≥ 10%	N	CD73 stroma H score <1	CD73 stroma H score ≥ 1
**Primary tumors**	45									
**De Novo**	30	30	17 (56.7%)	13 (43.3%)	29	21 (72.4%)	8 (27.6%)	30	7 (23.3%)	23 (76.7%)
**Archived**	15	15	7 (46.7%)	8 (53.3%)	15	9 (60%)	6 (40%)	15	6 (40%)	9 (60%)
**Metastatic lesions**	82									
**Breast**	13	13	9 (69.2%)	4 (30.8%)	12	9 (75%)	3 (25%)	13	4 (30.8%)	9 (69.2%)
**Brain**	1	1	1 (100%)	0 (0%)	1	1 (100%)	0 (0%)	1	0 (0%)	1 (100%)
**Chest Wall**	5	5	5 (100%)	0 (0%)	4	2 (50%)	2 (50%)	5	2 (40%)	3 (60%)
**Liver**	15	15	9 (60%)	6 (40%)	14	9 (64.3%)	5 (35.7%)	14	3 (21.4%)	11 (78.6%)
**Lung**	9	9	7 (77.8%)	2 (22.2%)	9	4 (44.4%)	5 (55.6%)	9	4 (44.4%)	5 (55.6%)
**Lymph node**	25	25	16 (64%)	9 (36%)	24	12 (50%)	12 (50%)	25	7 (28%)	18 (72%)
**Peritoneal**	1	1	1 (100%)	0 (0%)	1	1 (100%)	0 (0%)	1	1 (100%)	0 (0%)
**Skin**	8	8	7 (87.5%)	1 (12.5%)	8	8 (100%)	0 (0%)	8	4 (50%)	4 (50%)
**Uterus**	1	1	1 (100%)	0 (0%)	1	1 (100%)	0 (0%)	1	1 (100%)	0 (0%)
**Other**	2	2	2 (100%)	0 (0%)	2	2 (100%)	0 (0%)	2	0 (0%)	2 (100%)
**Not reported**	2	1	0 (0%)	1 (100%)	1	0 (0%)	1 (100%)	1	0 (0%)	1 (100%)

Supplementary Table 7. Stromal TILs and PD-L1 CPS categories between baseline and week 3.

	Arm A		Arm B		Total	
	With oleclumab		Without oleclumab			
**Stromal TILs**						
**Change in str-TILs between baseline and week 3**						
N	30		28		58	
Decrease ≥ 5%	6 (20.0%)		6 (21.4%)		12 (20.7%)	
Increase ≥ 5%	6 (20.0%)		9 (32.1%)		15 (25.9%)	
No change > or < 5%	18 (60%)		13 (46.4%)		31 (53.4%)	
**Change in str-TILs between baseline and week 3 according to clinical response**						
Clinical benefit	Yes	No	Yes	No	Yes	No
N	14	16	14	17	25	33
Decrease ≥ 5%	2 (14.3%)	4 (25.0%)	3 (21.4%)	3 (17.6%)	5 (20.0%)	7 (21.2%)
Increase ≥ 5%	5 (35.7%)	1 (6.3%)	5 (35.7%)	4 (23.5%)	10 (40.0%)	5 (15.1%)
No change > or < 5%	7 (50%)	11 (68.7%)	6 (42.8%)	10 (58.8%)	10 (40%)	21 (63.6%)
**PD-L1 CPS**						
**Change in CPS categories between baseline and week 3**						
N	31		30		61	
Decrease	4 (12.9%)		5 (16.7%)		9 (14.7%)	
Increase	19 (61.3%)		14 (46.6%)		33 (54.1%)	
No change	8 (25.8)		11 (36.7%)		19 (31.2%)	
**Change in CPS categories between baseline and week 3 according to clinical response**						
Clinical benefit	Yes	No	Yes	No	Yes	No
N	14	17	14	16	28	33
Decrease	3 (21.4%)	1 (5.9%)	3 (21.4%)	2 (12.5)	6 (21.4%)	3 (9.1%)
Increase	8 (57.1%)	11 (64.7%)	5 (35.7%)	9 (56.2)	13 (46.4%)	20 (60.6%)
No change	3 (21.5%)	5 (29.4%)	6 (42.9%)	5 (31.3)	9 (32.2%)	10 (30.3%)

Supplementary Table 8. CD73 histological score – exploratory cohort.

Characteristics	Arm A with oleclumab	Arm B without oleclumab	Total
**CD73 stroma histological score (H score)**			
N	55	55	110
<1	13 (23.6%)	10 (18.2%)	23 (20.9%)
≥1	42 (76.4%)	45 (81.9%)	87 (79.1%)
Mean (SD)	36.7 (57.8)	77.3 (81.4)	57.0 (73.9)
Median (IQR)	10 (1, 45)	40 (10, 45)	12.5 (1, 97.5)
**primary tumors**			
N	13	17	30
<1	2 (15.4%)	0 (0.0%)	2 (6.7%)
≥1	11 (84.6%)	17 (100.0%)	28 (93.3%)
**metastatic lesions**			
N	42	38	80
<1	11 (26.2%)	10 (26.3%)	21 (26.3%)
≥1	31 (73.8%)	28 (73.7%)	59 (73.7%)
**CD73 tumor histological score (H score)**			
N	55	55	110
<1	50 (90.9)	49 (89.1%)	99 (90.0%)
≥1	5 (9.1%)	6 (10.9%)	11 (10.0%)
Missing	8	9	17
Mean (SD)	3.3 (17)	11.1 (51.4)	7.1 (38.2)
Median (IQR)	0	0	0
**primary tumors**			
N	13	17	30
<1	12 (92.3%)	15 (88.2%)	27 (90.0%)
≥1	1 (7.7%)	2 (11.8%)	3 (10.0%)
**metastatic lesions**			
N	42	38	80
<1	38 (90.5%)	34 (89.5%)	72 (90.0%)
≥1	4 (9.5%)	4 (10.5%)	8 (10%)
