# Supplementary material for: Paclitaxel plus carboplatin and durvalumab with or without oleclumab for women with previously untreated locally advanced or metastatic triple-negative breast cancer: the randomized SYNERGY phase I/II trial
Source: Nat Commun. 2023 Nov 2;14:7018. doi: 10.1038/s41467-023-42744-y (PMC10622534; doi:10.1038/s41467-023-42744-y)
Supplement: Supplementary file 3 — Reporting Summary [file 41467_2023_42744_MOESM3_ESM.pdf]

nature portfolio

Corresponding author(s): Dr Laurence Buisseret

Last updated by author(s): Oct 19, 2023

Reporting Summary

Nature Portfolio wishes to improve the reproducibility of the work that we publish. This form provides structure for consistency and transparency in reporting. For further information on Nature Portfolio policies, see our Editorial Policies and the Editorial Policy Checklist.

Statistics

For all statistical analyses, confirm that the following items are present in the figure legend, table legend, main text, or Methods section.

n/a	Confirmed	
☐	☒	The exact sample size (n) for each experimental group/condition, given as a discrete number and unit of measurement
☒	☐	A statement on whether measurements were taken from distinct samples or whether the same sample was measured repeatedly
☐	☒	The statistical test(s) used AND whether they are one- or two-sided<br>*Only common tests should be described solely by name; describe more complex techniques in the Methods section.*
☐	☒	A description of all covariates tested
☐	☒	A description of any assumptions or corrections, such as tests of normality and adjustment for multiple comparisons
☐	☒	A full description of the statistical parameters including central tendency (e.g. means) or other basic estimates (e.g. regression coefficient) AND variation (e.g. standard deviation) or associated estimates of uncertainty (e.g. confidence intervals)
☐	☒	For null hypothesis testing, the test statistic (e.g. F, t, r) with confidence intervals, effect sizes, degrees of freedom and P value noted<br>*Give P values as exact values whenever suitable.*
☒	☐	For Bayesian analysis, information on the choice of priors and Markov chain Monte Carlo settings
☐	☒	For hierarchical and complex designs, identification of the appropriate level for tests and full reporting of outcomes
☒	☐	Estimates of effect sizes (e.g. Cohen's d, Pearson's r), indicating how they were calculated

Our web collection on statistics for biologists contains articles on many of the points above.

Software and code

Policy information about availability of computer code

Data collection	Open Clinica
Data analysis	SAS Entreprise Guide version 8.3; Graph Pad Prism version 9.5.1

For manuscripts utilizing custom algorithms or software that are central to the research but not yet described in published literature, software must be made available to editors and reviewers. We strongly encourage code deposition in a community repository (e.g. GitHub). See the Nature Portfolio guidelines for submitting code & software for further information.

Data

Policy information about availability of data

All manuscripts must include a data availability statement. This statement should provide the following information, where applicable:

- Accession codes, unique identifiers, or web links for publicly available datasets
- A description of any restrictions on data availability
- For clinical datasets or third party data, please ensure that the statement adheres to our policy

The raw and processed data generated in this study have been deposited at the Data Centre at Institut Jules Bordet in Brussels (Belgium) and can be made available upon approval of a research proposal. Any request for data (e.g., individual de-identified participant data, additional study documents including study protocol and/or statistical analysis plan) will be reviewed by the study team and should be addressed to Dr. Laurence Buisseret at laurence.buisseret@bordet.be. Restrictions may apply to requests from industry or for commercial purposes. The expected timeframe for response to access requests is 6 months. Once access has been granted the data will be available for 12 months (extendible upon approval).

Field-specific reporting

Please select the one below that is the best fit for your research. If you are not sure, read the appropriate sections before making your selection.

[x] Life sciences [ ] Behavioural & social sciences [ ] Ecological, evolutionary & environmental sciences

For a reference copy of the document with all sections, see nature.com/documents/nr-reporting-summary-flat.pdf

Life sciences study design

All studies must disclose on these points even when the disclosure is negative.

Sample size	127
Data exclusions	No data were excluded
Replication	Clinical trial : replicates not applicable
Randomization	Patients were randomized 1:1 between chemotherapy + durvalumab + oleclumab (Arm A) and chemotherapy + durvalumab (Arm B).
Blinding	Open-label study; no blinding

Reporting for specific materials, systems and methods

We require information from authors about some types of materials, experimental systems and methods used in many studies. Here, indicate whether each material, system or method listed is relevant to your study. If you are not sure if a list item applies to your research, read the appropriate section before selecting a response.

Materials & experimental systems

n/a	Involved in the study	
[ ]	[x]	Antibodies
[x]	[ ]	Eukaryotic cell lines
[x]	[ ]	Palaeontology and archaeology
[x]	[ ]	Animals and other organisms
[ ]	[x]	Human research participants
[ ]	[x]	Clinical data
[x]	[ ]	Dual use research of concern

Methods

n/a	Involved in the study	
[x]	[ ]	ChIP-seq
[x]	[ ]	Flow cytometry
[x]	[ ]	MRI-based neuroimaging

Antibodies

Antibodies used	PD-L1 (SP263) Rabbit Monoclonal primary antibody de VENTATA®: REF (catalogue number): 790-4905. LOT :H17857.<br>CD73 clone [EPR6115]) de ABCAM. REF: ab124725<br>GR3195327-2 (1.548mg/ml), YO092001PS
Validation	All commertial antibodies. PD-L1 is used in clinical practice. CD73 has been validated on positive and negative controls by a pathologist.

Human research participants

Policy information about studies involving human research participants

Population characteristics	The SYNERGY trial enrolled patients with advanced inoperable or metastatic TNBC, previously not treated. The main inclusion criteria were:<br>- female<br>- 18 years or aider<br>- with an Eastern Cooperative Oncology Group (ECOG) Performance Status of O or 1,<br>- at least one measurable lesion per RECIST vl.l.<br>Patients agreed ta provide tumor tissue for central assessment of PD-Ll and CD73 IHC status prior ta randomization for stratification and a second biopsy at week 3 for translational research purposes.
Recruitment	A total of 129 patients were randomized between June 2019 and June 2021 from 16 centers in Belgium and France, and 127 were evaluable for the primary endpoint, 63 in arm A and 64 in arm B.
Ethics oversight	This research complies with all relevant ethical regulations. The SYNERGY trial was approved by the following ethical committees/authorities: Comité d'éthique hospitalo-facultaire Erasme-ULB and Federal Agency for medicines and health products (Belgium), and Comité de protection des personnes Ile de France and Agence nationale de sécurité du medicament et des produits de santé (France).<br>The study design and conduct complied with all relevant regulations regarding the use of human study participants and was conducted in accordance with the criteria set by the Declaration of Helsinki. All patients signed written informed consent before inclusion.<br>The SYNERGY trial was registered on ClinicalTrials.gov (https://clinicaltrials.gov/) (ID: NCT03616886).

Note that full information on the approval of the study protocol must also be provided in the manuscript.

Clinical data

Policy information about clinical studies

All manuscripts should comply with the ICMJEguidelines for publication of clinical research and a completedCONSORT checklist must be included with all submissions.

Clinical trial registration	NCT03616886
Study protocol	Added in supplementary
Data collection	Data collection was performed between June 2019 and January 2023
Outcomes	The primary endpoint waw clinical benefit (CB). CB is defined as a patient who achieved CR or PR or demonstrated SD at 24 weeks from the 1st dose of study drug administration based on RECIST v1.1.<br>Secondary endpoints were :<br>- Objective Response. OR is defined as a patient (in the intent-to treat population) who achieved a CR ou PR as best overall response (BOR) based on RECIST v1.1.<br>- Duration of Response. DOR is defined as the time from documentation of first tumour response to disease progression based on RECIST v1.1.<br>- Progression Free Survival. PFS is defined as the time from 1st study drug administration to the first documented disease progression based on RECIST v1.1 or death due to any cause, whichever occurs first. (Subjects who are alive and progression free at the time of analysis will be censored at the time-point of their last tumour assessment by imaging).<br>- Overall Survival. OS is defined as the time from 1st study drug administration to death due to any cause. (Subject whitout documented death at the time of the analysis will be censored at the date of the last follow-up).<br>- Frequency, duration and severity of AEs based on CTCAE 5.0.
